# Insanity as a Defence in the Criminal Courts—III

**Published:** 1908-11-07

**Authors:** 


### Medico-Legal Points.

### INSANITY AS A DEFENCE IN THE CRIMINAL COURTS—III.

In the case of *Rex* v. *Offord* (5 C. and P. 168) in 1831, Lord Lyndhurst charged the jury that they must be satisfied that the accused "did not know, when he committed the act, what the effect of it, if fatal, would be with reference to the crime of murder. The question was, did he know that he was committing an offence against the laws of God and Nature? He then referred to Bellingham's case, and agreed with Lord Mansfield's opinion. Offord had delusional insanity of a most confirmed type, believing that the inhabitants of Hadleigh were in league against his life. Under the influence of these delusions he would abuse strangers in the streets; and he carried a list of about fifty names, entitled "List of Hadleigh conspirators against my life." He shot and killed a man named Chisnall, whose name was on this list. He was acquitted "on the ground of insanity."

The two cases of Bellingham and Offord present a striking example of the inconsistency of the law. Both these men were delusional lunatics of exactly the same type; for they both suffered from delusions of persecution, which render such lunatics the most dangerous of all the insane. The jury in each case was charged in practically the same language, and yet Bellingham was hanged and Offord was acquitted.

In the next case of importance, however—that of *Reg* v. *Oxford* (9 c. and p. 525)—a distinct advance came about by a proper scientific statement of criminal insanity. Lord Chief Justice Denman charged the jury: "If some controlling disease was, in truth, the acting power within him, which he could not resist, then he will not be responsible." This was an unexceptionable statement from a scientific standpoint, and it is unfortunate that it was not allowed to stand without further elucidation. It would have constituted an exemplary definition for all future ages, and would have been to the everlasting credit of the distinguished judge who uttered it. But Lord Denman, with that tendency to refine and elaborate which is so characteristic of the legal mind, did not seem satisfied that it exhausted the law of the subject, for he continued: "The question is whether the prisoner was labouring under that species of insanity which satisfies you that he was quite unaware of the nature, character, and consequences of the act he was committing: or, in other words, whether he was under the influence of a diseased mind, and was really unconscious, at the time he was committing the act, that it was a crime." He also used the expression whether "he was insane at the time when the act was done; whether the evidence given proves a disease of the mind, as of a person quite incapable of distinguishing right from wrong."

It is evident all through this charge that Lord Denman's mind grasped the central idea—the essential issue in all such cases—and grasped it much more clearly than any of his predecessors had done. He reverts again and again to the simple question whether the prisoner was "insane." It was only when he attempted to qualify that question with the consideration of the "knowledge of right and wrong" and of the "knowledge of the nature of the act" that he spoiled a good definition, and lost a great opportunity. That the jury saw the point, however, is evident from the fact that they found Oxford "not guilty, on the ground of insanity." They did not find that the prisoner did not know the difference between right and wrong, or that he did not know that it was a crime to shoot at the Queen, for their common sense must have told them that he probably knew both these things. But they took Lord Denman at his word, and found merely that Oxford had a "disease of the mind."

M'Naghten, like Hadfield and Bellingham, was a delusional lunatic. His delusions were of the persecutory type; he believed that he was harassed by enemies, and, including Mr. Drummond, the private secretary of Sir Robert Peel, among these enemies, he had shot and killed him in the street. He was successfully defended, and was acquitted on the ground of insanity; but the verdict was unpopular, and the House of Lords, responsive to popular criticism, addressed a request to the judges for an authoritative statement of the law on the subject of insanity as a defence for crime. The prisoner was tried in 1843 (10 Cl. and F. 200). The evidence was that M'Naghten was suffering with "morbid delusions," and that persons so suffering "might have a moral perception of right and wrong, but that, in the case of the prisoner, it was a delusion which carried him away beyond the power of his own control, and left him no such perception; and that he was not capable of exercising any control over acts which had connection with his delusion; that it was of the nature of the disease with which the prisoner was affected to go on gradually until it had reached a climax, when it burst forth with irresistible intensity; that a man might go on for years quietly, though at the same time under its influences, but would all at once break out into the most extravagant and violent paroxysms."

The above is the statement of M'Naghten's case, as submitted to the House of Lords. It is to be noted that it is a clear statement of a case of delusional insanity; only in skeleton outlines, to be sure, but still giving the essential points in such a case. It meets the question of right and wrong, and disposes of it. It also claims that such lunatics have no control over their delusions. This is practically true; but it is not equally true in all cases. Lunatics vary in this respect, but in a criminal act by a delusional lunatic the inference is that he was controlled by his delusion. In M'Naghten's case this was the vital scientific point—*i.e.* the fact that he was controlled by his delusions; not the question whether he had the ability to distinguish between right and wrong in the abstract, or even in the concrete. With reference to his particular act, however, his moral perceptions were doubtless obscured by his delusion.

Lord Chief Justice Tindal, in his charge to the jury, had said: "The question to be determined is whether, at the time the act in question was committed, the prisoner had or had not the use of his understanding as to know that he was doing a wrong or wicked act. If the jurors should be of opinion that the prisoner was not sensible at the time he committed it that he was violating the laws both of God and man, then he would be entitled to a verdict in his favour; but if, on the contrary, they were of opinion that when he committed the act he was in a sound state of mind, then their verdict must be against him."

M'Naghten had been found "not guilty, on the ground of insanity"—a righteous verdict, legally and scientifically worded. But the case was brought up for discussion in the House of Lords, and on June 19, 1843, a series of questions were propounded to the judges, as follows:

"1st. What is the law respecting alleged crimes committed by persons afflicted with insane delusion in respect of one or more particular subjects or persons, as, for instance, when, at the time of the commission of the alleged crime, the accused knew he was acting contrary to law, but did the act complained of with a view, under the influence of insane delusion, of redressing or revenging some supposed grievance or injury, or of producing some supposed public benefit?"

"2nd. What are the proper questions to be submitted to the jury when a person alleged to be affected with insane delusion respecting one or more particular subjects or persons is charged with the commission of a crime (murder, for example) and insanity is set up as a defence?"

"3rd. In what terms ought the question to be left to the jury as to the prisoner's state of mind when the act was committed?"

"4th. If a person under an insane delusion as to existing facts commits an offence in consequence thereof, is he thereby excused?"

"5th. Can a medical man, conversant with the disease of insanity, who never saw the prisoner previously to the trial, but who was present during the whole trial and the examination of all the witnesses, be asked his opinion as to the state of the prisoner's mind at the time of the commission of the alleged crime, or his opinion whether the prisoner was conscious, at the time of doing the act, that he was acting contrary to law, or whether he was labouring under any, and what, delusion at the time?"

Stripped of redundancy these questions mean simply this: 1st. What is the law respecting the alleged crimes of delusional lunatics? 2nd. What is the question at issue when a delusional lunatic commits an alleged crime? The third question practically repeats the second. The fourth question practically repeats the first. The second question also very nearly repeats the first. There probably never was a series of questions embodying one simple point of inquiry clothed in such redundancy and reiteration. The point at issue simply was—What was the English law, in 1843, concerning delusional lunatics who committed crimes under the influence of their delusions? This was the point of inquiry, and this point of inquiry, one would suppose, was the proper and only one to be submitted to the jury with reference to the case on trial. But in striving after refinements of definition, this simple inquiry was obscured in a crowd of involved interrogations, which, when analysed, can mean only one thing—namely, What is the English law respecting delusional lunatics who commit crimes? The fifth question was entirely apart from the main inquiry, and refered merely to the privilege of an expert giving his opinion on the evidence at the trial.

The answers given by the judges to these questions suggest the suspicion that both the questions and the answers were devised by the same mind or minds. They are written in the same redundant and involved style, and the same preconceived opinions are apparent in them. The answers of the judges, delivered by Lord Chief Justice Tindal, who had presided at the trial of M'Naghten, embodied the following opinion. A delusional lunatic is responsible if he knew "that he was acting contrary to law. . . . That to establish a defence on the ground of insanity, it must be clearly proved that, at the time of the committing of the act, the party accused was labouring under such a defect of reason, from disease of the mind, as not to know the nature and quality of the act he was doing, or, if he did know it, that he did not know he was doing what was wrong." And this knowledge was further qualified as pertaining, not to the abstract, but to "the very act with which he is charged"; and then certain refinements were made as to the prisoner's knowledge of the "law of the land," which the law presumes all men to have. A delusional lunatic "must be considered in the same situation as to responsibility as if the facts with respect to which the delusion exists were real." That is, if the lunatic kills another man in supposed self-defence, even though acting under a delusion, he is exempt; but if his delusion is that the other man has simply done injury to his character and fortune, he is liable to punishment.

